# Network-Assisted Investigation of Combined Causal Signals from Genome-Wide Association Studies in Schizophrenia

**DOI:** 10.1371/journal.pcbi.1002587

**Published:** 2012-07-05

**Authors:** Peilin Jia, Lily Wang, Ayman H. Fanous, Carlos N. Pato, Todd L. Edwards, The International Schizophrenia Consortium, Zhongming Zhao

**Affiliations:** 1 Department of Biomedical Informatics, Vanderbilt University School of Medicine, Nashville, Tennessee, United States of America; 2 Department of Psychiatry, Vanderbilt University School of Medicine, Nashville, Tennessee, United States of America; 3 Department of Biostatistics, Vanderbilt University School of Medicine, Nashville, Tennessee, United States of America; 4 Department of Psychiatry and Virginia Institute for Psychiatric and Behavior Genetics, Virginia Commonwealth University, Richmond, Virginia, United States of America; 5 Washington VA Medical Center, Washington, D.C., United States of America; 6 Department of Psychiatry, Georgetown University School of Medicine, Washington, D.C., United States of America; 7 Department of Psychiatry, Keck School of Medicine of the University of Southern California, Los Angeles, California, United States of America; 8 Center for Human Genetics Research, Vanderbilt University School of Medicine, Nashville, Tennessee, United States of America; 9 Division of Epidemiolgy, Department of Medicine, Vanderbilt University School of Medicine, Nashville, Tennessee, United States of America; 10 Department of Cancer Biology, Vanderbilt University School of Medicine, Nashville, Tennessee, United States of America; Harvard Medical School, United States of America

## Abstract

With the recent success of genome-wide association studies (GWAS), a wealth of association data has been accomplished for more than 200 complex diseases/traits, proposing a strong demand for data integration and interpretation. A combinatory analysis of multiple GWAS datasets, or an integrative analysis of GWAS data and other high-throughput data, has been particularly promising. In this study, we proposed an integrative analysis framework of multiple GWAS datasets by overlaying association signals onto the protein-protein interaction network, and demonstrated it using schizophrenia datasets. Building on a dense module search algorithm, we first searched for significantly enriched subnetworks for schizophrenia in each single GWAS dataset and then implemented a discovery-evaluation strategy to identify module genes with consistent association signals. We validated the module genes in an independent dataset, and also examined them through meta-analysis of the related SNPs using multiple GWAS datasets. As a result, we identified 205 module genes with a joint effect significantly associated with schizophrenia; these module genes included a number of well-studied candidate genes such as *DISC1*, *GNA12*, *GNA13*, *GNAI1*, *GPR17*, and *GRIN2B*. Further functional analysis suggested these genes are involved in neuronal related processes. Additionally, meta-analysis found that 18 SNPs in 9 module genes had *P*_meta_<1×10^−4^, including the gene *HLA-DQA1* located in the MHC region on chromosome 6, which was reported in previous studies using the largest cohort of schizophrenia patients to date. These results demonstrated our bi-directional network-based strategy is efficient for identifying disease-associated genes with modest signals in GWAS datasets. This approach can be applied to any other complex diseases/traits where multiple GWAS datasets are available.

## Author Summary

The recent success of genome-wide association studies (GWAS) has generated a wealth of genotyping data critical to studies of genetic architectures of many complex diseases. In contrast to traditional single marker analysis, an integrative analysis of multiple genes and the assessment of their joint effects have been particularly promising, especially upon the availability of many GWAS datasets and other high-throughput datasets for numerous complex diseases. In this study, we developed an integrative analysis framework for multiple GWAS datasets and demonstrated it in schizophrenia. We first constructed a GWAS-weighted protein-protein interaction (PPI) network and then applied a dense module search algorithm to identify subnetworks with combinatory disease effects. We applied combinatorial criteria for module selection based on permutation tests to determine whether the modules are significantly different from random gene sets and whether the modules are associated with the disease in investigation. Importantly, considering there are many complex diseases with multiple GWAS datasets available, we proposed a discovery-evaluation strategy to search for modules with consistent combined effects from two or more GWAS datasets. This approach can be applied to any diseases or traits that have two or more GWAS datasets available.

## Introduction

Genome-wide association (GWA) studies have, during the past decade, become a powerful tool to study the genetic components of complex diseases [1]. Although an increasing number of genes/markers have been uncovered in GWA studies, which have provided us important insights into the underlying genetic basis of complex diseases such as schizophrenia [2], [3], [4], it has also become evident that many genes are weakly or moderately associated with the diseases. Most of these variants have been missed in single marker analysis, as investigators typically employ a genome-wide significance cutoff *P*-value of 5×10^−8^. Alternatively, the gene set analysis (GSA) of GWAS datasets provides ways to simultaneously examine groups of functionally related genes for their combined effects and thus have improved power and interpretability [5].

Many GSA methods have been reported to date, such as the gene set enrichment analysis [6], the adaptive rank-truncated product [7], the gene set ridge regression in association studies (GRASS) [8], etc. Most of these methods were designed to use pre-defined gene sets such as the KEGG database [9] or the Gene Ontology (GO) annotations [10]. Alternatively, studies are emerging by incorporating protein-protein interaction (PPI) networks into GWAS analysis. Baranzini *et al.* [11] first adopted a network-based method that was initially designed for gene expression data [12] to analyze the GWAS data for multiple sclerosis. Recently, Rossin *et al.* [13] developed the Disease Association Protein-Protein Link Evaluator (DAPPLE); it tests whether genes that are located at association loci in a GWAS dataset are significantly connected via PPIs. We have also developed the dense module search (DMS) method [14], which overlays the gene-wise *P* values from GWAS onto the PPI network and dynamically searches for subnetworks that are significantly enriched with the association signals.

The advantages of network-based analysis of GWAS data in comparison with the standard GSA methods lie in many aspects. First, most GSA methods test on pre-defined gene sets, which heavily rely on *a priori* knowledge and are incomplete. For example, the popular KEGG database has pathway annotations covering only ∼5,000–5,500 genes [15], accounting for less than 30% of the genes in GWAS datasets. In contrast, the annotations of PPI data cover a much larger proportion of human proteins. For example, a recent integrative analysis of PPI data from multiple sources has reconstructed the human PPI network by recruiting ∼12,000 proteins and ∼60,000 protein interaction pairs with experimental evidence [16]. There are other assembled PPI datasets that include both experimentally supported and computationally predicted interactions; thus, they could annotate even more proteins and interactions [17], [18]. Second, the standard GSA methods are typically based on canonical definitions of pathways or functional categories, but the association signals from GWAS might converge on only part of the pathway [19]. In such cases, analysis of the whole pathway as a unit would reduce the power. On the other hand, network-assisted methods allow for the definition of *de novo* gene sets by dynamically searching for interconnected subnetworks in the whole interactome and, thus, can effectively alleviate the limitation of the fixed size in pathway analysis.

Despite these advantages, there are challenges in the application of network-based approaches to GWAS data. For example, the methods for defining or searching subnetworks vary greatly. While it is impractical to examine all possible subnetworks due to the intensive computing burden, different methods or algorithms may identify substantially different subnetworks [20], making it difficult to decide in real application. Additionally, network-based analysis could be confounded by nodes with high degrees (i.e., the number of interactors of each node in the network), although these nodes constitute only a small proportion according to the framework of power-law distribution [21]. One example is TP53, which interacts with several hundreds of other proteins in the whole PPI network. The existence of such hub nodes with strong interaction in the network may help them more likely to be selected in searching subnetworks and, thus, overwhelm the resultant subnetworks. Appropriate adjustments are needed.

In this study, we aim to search for modules that are significantly enriched with association signals in human PPI network weighted by GWAS signals. We take advantage of our recently developed dense module search (DMS) algorithm [14] to conduct module searching and construction. Based on this, we introduced statistical evaluations of the modules identified by DMS, including a significance test based on module scores, a weighted resampling method to adjust potential biased in GWAS data (e.g., caused by gene length or SNP density), a topologically matched randomization process to adjust the bias in network (e.g., the high degree nodes), and a permutation test to determine the disease association of the modules. In addition, we propose a bi-directional framework to search for consistent association signals from multiple GWAS datasets available for one specific disease or trait. Specifically, two GWAS datasets were analyzed in parallel: one is assigned as a discovery dataset and another as an evaluation dataset, and vice versa. This strategy provides robust results with partial validation — only the modules that were consistently highly scored would be selected for further validation and functional assessment. We demonstrated it in schizophrenia using two major GWAS datasets for module identification, and incorporated a third dataset to independently replicate the results. Finally, we performed a meta-analysis of the markers that were mapped in the module genes. We identified 18 SNPs in 9 module genes that are of particular interests (*P*_meta_<1×10^−4^).

## Results

### An overview of the network framework for GWAS

**Figure 1:**
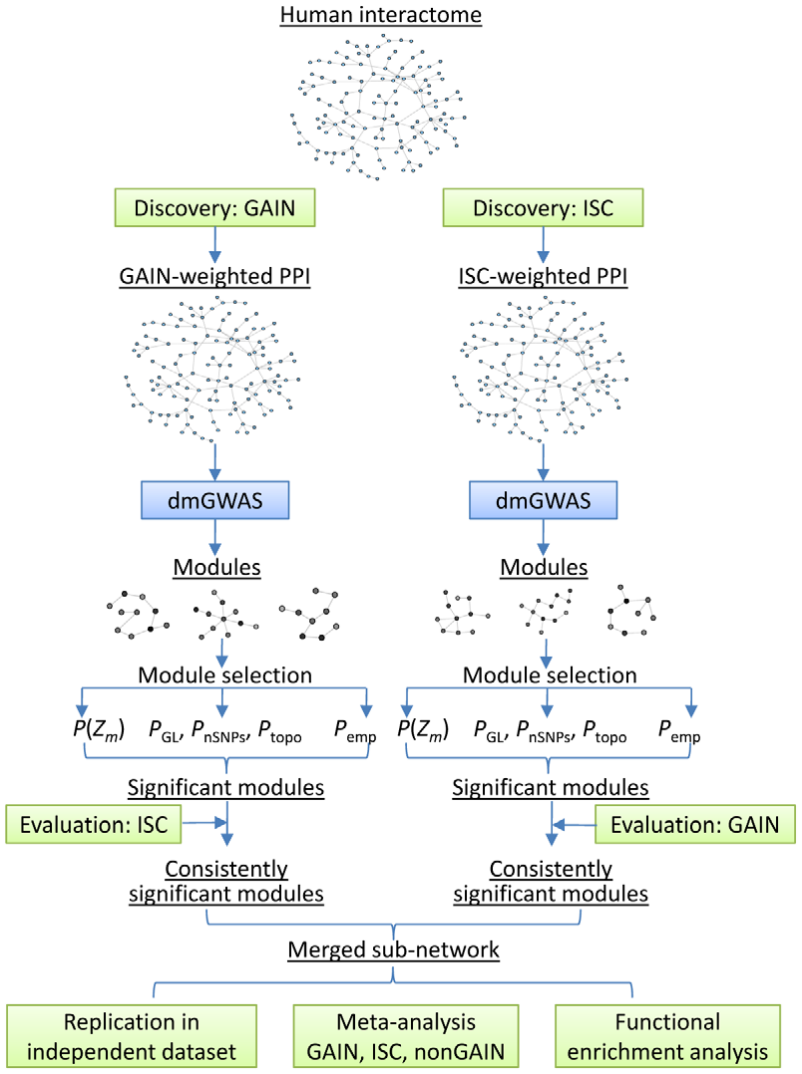
Materials and MethodscMaterials and Methodsgenes for schizophrenia.

We incorporated two case-control GWAS datasets for schizophrenia in this study for module search: the International Schizophrenia Consortium (ISC) study and the Genetic Association Information Network (GAIN) dataset. A third dataset, the Molecular Genetics of Schizophrenia (MGS) - nonGAIN dataset, was included in the validation stage by bringing independent samples for disease association test. Each of the three datasets was preprocessed and quality controlled, with none observed to have substantial population stratification. As shown in Figure 1, we started with the GAIN dataset for module discovery, followed by module evaluation using the ISC dataset. In the parallel thread, the ISC dataset was used for constructing modules and the GAIN dataset for evaluation. In both threads, a series of significance tests were performed, each of which aims to build null distributions for different purposes and adjust specific biases. The modules that passed the filtering criteria in both datasets were selected and merged. Module genes were collected and considered as schizophrenia candidate genes, whose association signals were further examined in an independent GWAS dataset, the nonGAIN dataset, as well as, by meta-analysis using three GWAS datasets (ISC, GAIN, and nonGAIN).

More specifically, our algorithm for multiple GWAS datasets includes the following steps.

Step 1. Candidate module search in one GWAS dataset. The gene-wise *P* values from the GWAS results were converted to *z*-scores and overlaid to the background human interactome (the whole PPI network), with each node being weighted by the *z*-score of the encoding genes. For each node in the network, DMS is performed to generate a best module, i.e., with the largest module score, *Z_m_* (see Materials and Methods). We performed this module construction step for each GWAS dataset using the R package, *dmGWAS*, which implements the original DMS algorithm [14], and the default parameters were used.

Step 2. Module assessment. We provide three types of significance tests to assess the candidate modules: (1) the significance test based on module scores (*P*(*Z_m_*)); (2) the evaluation of module scores in the context of various biases (*P*_GL_, *P*_nSNPs_, and *P*_topo_); and (3) the permutation test by shuffling disease labels in the GWAS datasets (*P*_emp_). Detailed information can be found in the Materials and Methods section.

Step 3. Module selection. In practice, several thousands of modules are likely to be constructed, corresponding to the thousands of genes used as seed; thus, further selection for top modules is needed. In a single GWAS-weighted module search process, we employed the following combinatorial criteria to select modules: (1) *P*(*Z_m_*)<0.05; (2) *P*_GL_<0.05, *P*_nSNPs_<0.05, and *P*_topo_<0.05; and (3) *P*_emp_<0.05. When there are two GWAS datasets available for the same disease or trait, we propose to use one dataset serving as discovery (*discov*) and the other as evaluation (*eval*), and vice versa (Figure 1). This allows us to select the most reliable modules with enriched association signals from more than one study. For each module generated by the discovery dataset, we also computed the corresponding *P*(*Z_m_*_(*eval*)_) using the same set of genes (i.e., in the same module) with gene weights based on the evaluation GWAS dataset, as well as *P_emp(eval)_* by shuffling the case/control labels in the evaluation GWAS dataset. Modules were selected if they have *P*(*Z_m_*_(*eval*)_)<0.05 and *P_emp(eval)_*<0.05.

### Dense module search for schizophrenia

**Figure 2:**
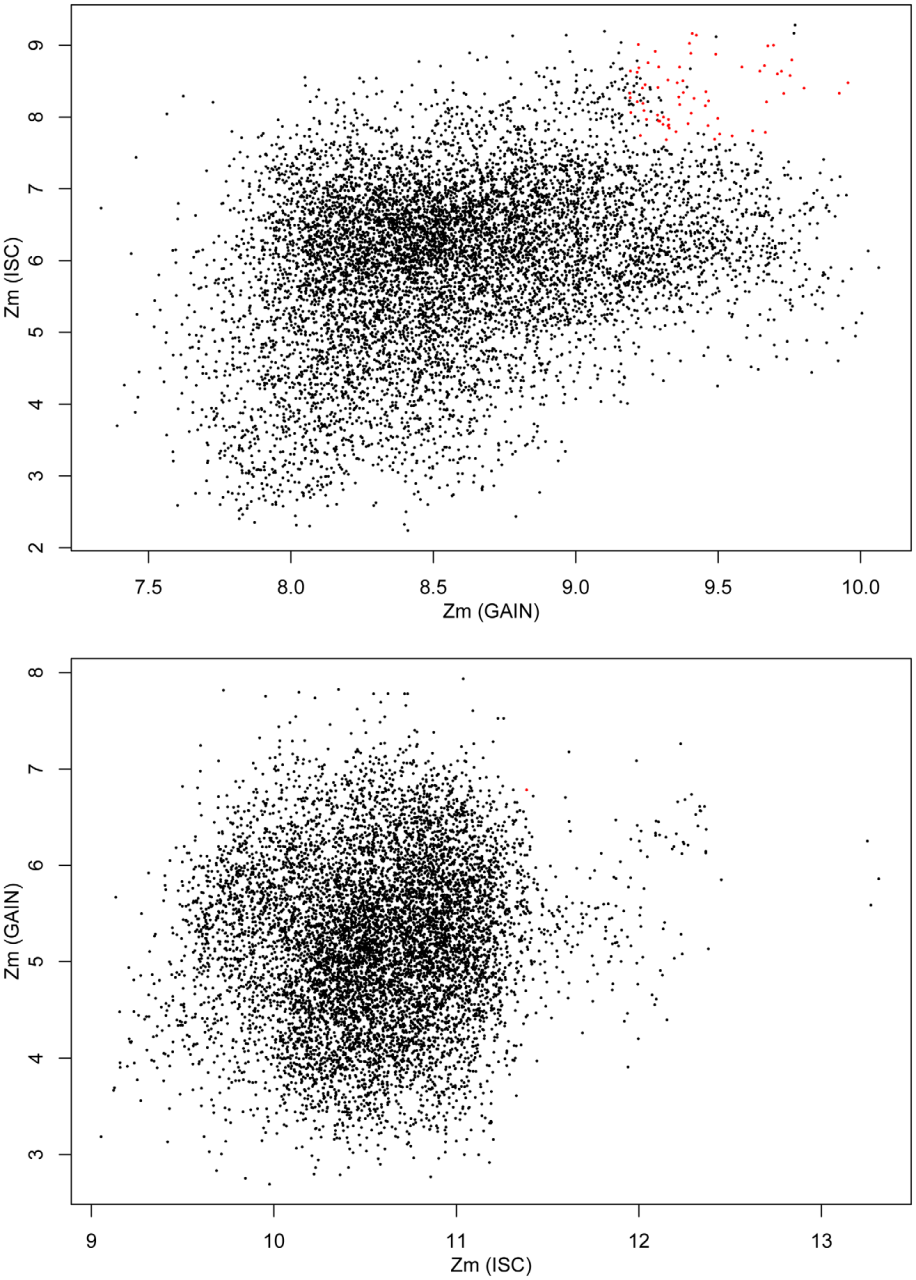
Distribution of module scores (*Z_m_*) from two GWAS datasets. Each circle in the plot represents a module. The circles in red indicate those selected modules (see text). X-axis: module scores from the discovery GWAS dataset. Y-axis: module scores from the evaluation GWAS dataset.

Using GAIN as the discovery dataset, we identified a total of 8,739 modules (Figure 2A). The module size ranged between 5 and 17, with a median value of 11 (Figure S2). A total of 935 modules passed the combinatorial criteria, i.e., (1) *P*(*Z_m_*)<0.05; (2) *P*_GL_<0.05, *P*_nSNPs_<0.05, and *P*_topo_<0.05; and (3) *P*_emp_<0.05. Among them, 71 modules were also significant in the ISC evaluation dataset (*P*(*Z_m_*_(*eval*)_)<0.05). Furthermore, 68 out these 71 modules passed the permutation test in the evaluation dataset (*P*_emp(eval)_<0.05). They were denoted as the final list of modules.

Similarly, using ISC as the discovery dataset, we identified 8,899 modules (Figure 2B), with the module size ranging between 5 and 18 and a median value of 11 (Figure S2). A total of 259 modules passed the combinatorial criteria. However, only one module was significant when adding the GAIN dataset for evaluation, involving 11 genes. We then merged the two lists to build a PPI subnetwork, which consisted of 205 module genes (Figure S3).

### Module genes as candidates for schizophrenia

A substantial proportion of the 205 module genes had nominally significant *P* values (defined as *P*<0.05 without multiple testing correction) in the corresponding GWAS dataset: 139 module genes (67.80%) had *P*_GAIN_<0.05, and 125 module genes (60.98%) had *P*_ISC_<0.05. The remaining module genes with non-significant *P* values were recruited in the top modules due to their physical interactions with the nominally significant genes in the PPI network, as DMS aims to identify joint effects of a set of schizophrenia genes in the context of the PPI network. In summary, 97 of the 205 genes (47.32%) were nominally significant in both the GAIN and ISC datasets, and 167 (81.46%) were nominally significant in either dataset.

Further comparison of these genes with previous association studies in the SZGene database [22] (as of January 26, 2011) showed that 31 (15.12%) of the module genes had been studied for association with schizophrenia. The SZGene database manually curates the association results from previously published association studies as well as recent GWAS findings. Among these 31 genes, 16 had at least one positive association study in previous work. Eighteen of these 31 genes (58.06%) were nominally significant (gene-wise *P* value<0.05) in both the GAIN and ISC datasets, while 26 (83.87%) had nominal significance in either dataset. These proportions were similar to those evaluated for the whole 205 module genes above. In contrast, the corresponding proportions of nominally significant genes in whole GWAS datasets were much lower (16.43% genes with nominal significance in both datasets and 55.77% in at least one dataset), indicating that the identified module genes were closer to genes known to be associated with schizophrenia.

### Replication in an independent GWAS dataset

We further evaluated the 205 module genes in an independent GWAS dataset, the nonGAIN dataset. First, we assessed whether the module genes contain a proportion of significant genes than randomly expected. This was done through weighted resampling while controlling the potential biases of gene length and SNP density in the nonGAIN dataset. Representing each module gene by the smallest *P* value among the SNPs located in its gene region, we denote the gene as significant if its nominal *P* value was less than 0.05. The 205 module genes were pooled together and denoted as one gene set, in which we found 76 genes were observed to be nominally significant in the nonGAIN dataset. We executed the weighted resampling process by 10,000 times, and built a null distribution of the number of significant genes given the number of module genes. This process was executed in the same way as the second significance test in module assessment. The details can be found in the Materials and Methods section, as well as in previous study [23]. The empirical *P* for the module gene set was 0.002 when adjusting gene length, and 0.003 when adjusting SNP density, indicating that these genes are not expected from random cases.

Second, we assessed the module genes in nonGAIN through resampling of SNPs. The 205 module genes had a total of 15,548 SNPs in the nonGAIN dataset. In each resample, we randomly selected the same number of SNPs (i.e., 15,548 SNPs) out of all the SNPs genotyped in the nonGAIN dataset, and recorded the number of significant SNPs, which were again defined as those whose nominal *P* values<0.05. We repeated this process by 10,000 times and counted the number of resample processes having more significant SNPs than that of the real case. This analysis resulted in an empirical *P* value of 0.022, indicating that the SNPs harbored in these module genes contained a higher proportion of nominally significant SNPs than randomness.

Note that the nonGAIN dataset is independent of the GAIN and ISC datasets we used to discover the module genes. Therefore, these results provide an independent replication of our module genes and showed that they are significantly enriched with association signals to schizophrenia.

### Meta-analysis

There were 15,252 SNPs in the genomic regions of the 205 module genes that were genotyped in all three GWAS datasets. Using the inverse-variance weighted meta-analysis method and heterogeneity test, we identified a total of 1032 SNPs having nominal significance (*P*_meta_<0.05) after removing substantial heterogeneity (*P*_heterogeneity_<0.05).

To determine whether the module genes contain a proportion of significant SNPs higher than expected by chance, we randomly sampled SNP sets with the same number of SNPs mapped to module genes (i.e., 15,252) and computed the proportion of significant SNPs (defined as those with *P*_meta_<0.05). Repeating the random process by 1000 times, we computed the empirical *P* value by *P*_emp_ = 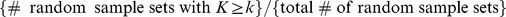, where *K* is the number of significant SNPs with *P*_meta_<0.05 in a random set, and *k* is the number in the real case, i.e., *k* = 1032. This random process showed that the module genes contains a significantly higher proportion of significant SNPs (*P*_emp_<0.001), further proving the enriched signal in the module genes.

**Figure 3:**
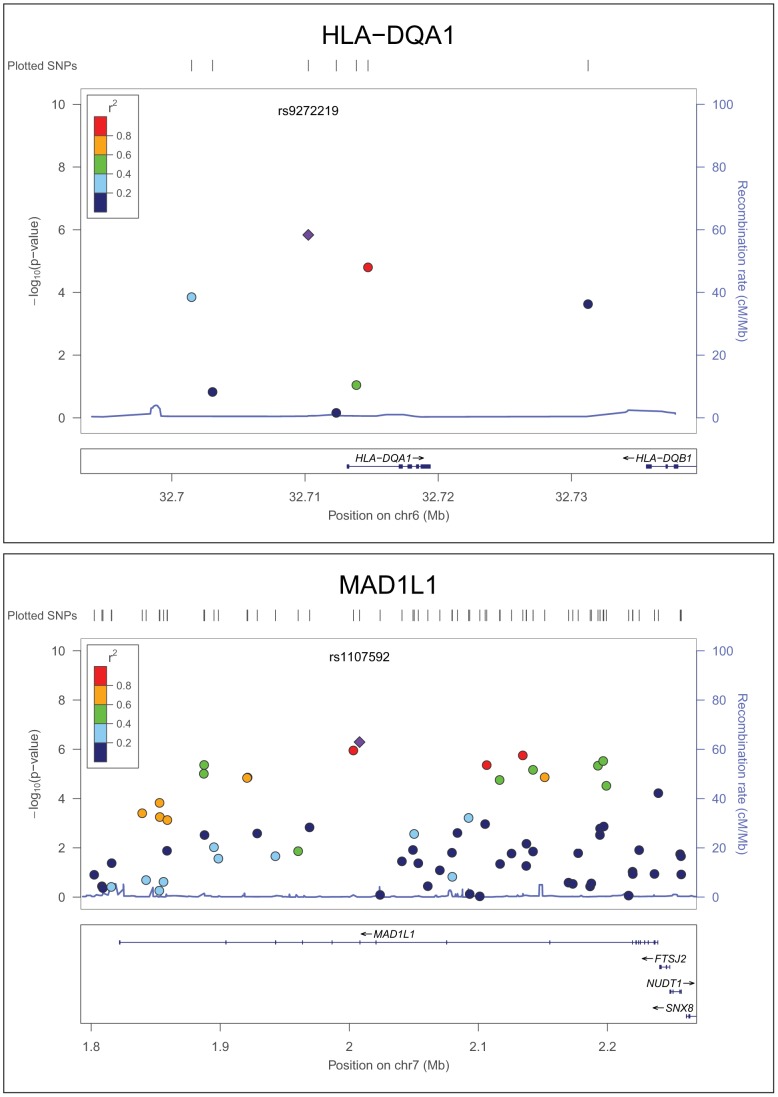
Meta-analysis results of the two most significant genes. Figures were generated using the LocusZoom online tool. X-axis is the genome coordinate. Y-axis is the -log*P*_meta_ values. Each point represents a SNP. The color of points is according to their level of linkage disequilibrium (LD) with the index SNPs. In this case, the index SNP is the most significant one in each panel. The LD measure is r^2^ based on the HapMap CEU population (release 22).

Among the significant module SNPs by meta-analysis, 18 SNPs in 9 genes were shown to have *P*_meta_<1×10^−4^ (Table 1). The most significant module SNPs were located in the gene *HLA-DQA1*, followed by *MAD1L1* (Table 1, Figure 3). There are two SNPs in *HLA-DQA1* with *P*_meta_<1×10^−4^: rs9272219 (*P*_meta_ = 1.46×10^−6^) and rs9272535 (*P*_meta_ = 1.58×10^−5^). Both were in the top list reported in a previous combined analysis of three GWAS datasets for schizophrenia [2], [3], [4], which included all the GWAS datasets we used here plus the SGENE dataset [4], to which we do not have access currently. The combined *P* value in the previous work [4] was *P*_comb_ = 6.9×10^−8^ for rs9272219 and *P*_comb_ = 8.9×10^−8^ for rs9272535. Both SNPs are located in the MHC region chr6: 27,155,235–32,714,734, a region that was reported to harbor a genome-wide significant association signal for schizophrenia [2]. Another gene, *MAD1L1*, has six SNPs with small *P*_meta_ values (4.30×10^−6^∼6.01×10^−5^, Table 1). *MAD1L1* is a long gene (∼417 kb) and has 70 overlapped SNPs examined in the meta-analysis. We further examined whether these 6 SNPs are located in the same LD block. Using the HapMap3 CEU data (http://www.hapmap.org/, release R2), we found that these SNPs were located in 4 blocks, suggesting that they might represent independent association signals.

**Table 1:** Results of meta-analysis using GAIN, nonGAIN, and ISC GWAS datasets (*P*_meta_<1×10^−4^ and *P*_heterogeneity_≥0.05).

SNP ID	Module Genes	Chr.	Position	Allele	*P*_meta_	Beta	s.e.	*P*_GAIN_	*P*_nonGAIN_	*P*_ISC_	*P*_heterogeneity_
rs9272219	*HLA-DQA1*	6	32710247	T/G	1.46×10^−6^	−0.15	0.03	0.06	0.06	1.58×10^−5^	0.76
rs10244946	*MAD1L1*	7	1887594	A/G	4.30×10^−6^	−0.16	0.03	1.81×10^−4^	0.18	2.36×10^−3^	0.27
rs3778994	*MAD1L1*	7	2142381	A/C	6.79×10^−6^	−0.15	0.03	4.54×10^−4^	0.61	1.20×10^−4^	0.07
rs10275045	*MAD1L1*	7	1887352	T/C	9.79×10^−6^	−0.13	0.03	1.44×10^−4^	0.20	3.61×10^−3^	0.16
rs4721190	*MAD1L1*	7	1921258	A/G	1.39×10^−5^	−0.15	0.03	3.07×10^−4^	0.17	6.42×10^−3^	0.32
rs2056480	*MAD1L1*	7	1920827	A/G	1.44×10^−5^	−0.12	0.03	4.15×10^−5^	0.31	5.69×10^−3^	0.07
rs9272535	*HLA-DQA1*	6	32714734	A/G	1.58×10^−5^	−0.16	0.04	0.07	0.07	8.27×10^−5^	0.41
rs3132649	*RPP21,TRIM39*	6	30429036	A/G	1.64×10^−5^	−0.20	0.05	0.01	0.46	6.46×10^−7^	0.00
rs10224497	*MAD1L1*	7	2116493	G/A	1.75×10^−5^	−0.14	0.03	4.32×10^−5^	0.91	8.46×10^−4^	0.02
rs741326	*CD207,CLEC4F*	2	70912343	G/A	2.65×10^−5^	−0.12	0.03	0.31	0.09	4.14×10^−5^	0.46
rs12646184	*SMARCAD1*	4	95402239	T/C	3.21×10^−5^	0.12	0.03	2.85×10^−5^	0.11	0.03	0.05
rs2071278	*AGER, NOTCH4*	6	32273422	G/A	3.23×10^−5^	−0.16	0.04	0.10	0.98	2.78×10^−6^	0.06
rs2664871	*SMARCAD1*	4	95365304	T/C	4.69×10^−5^	0.12	0.03	3.89×10^−5^	0.12	0.04	0.05
rs172531	*RERE*	1	8418177	G/A	5.62×10^−5^	0.12	0.03	0.01	0.75	4.03×10^−5^	0.06
rs2087170	*SMARCAD1*	4	95381983	G/T	5.83×10^−5^	0.14	0.03	5.59×10^−5^	0.10	0.12	0.11
rs3757440	*MAD1L1*	7	2239462	G/A	6.01×10^−5^	−0.14	0.04	6.41×10^−4^	0.56	2.35×10^−3^	0.14
rs301791	*RERE*	1	8390959	T/A	6.45×10^−5^	0.12	0.03	4.88×10^−3^	0.78	9.90×10^−5^	0.06
rs301801	*RERE*	1	8418532	C/T	6.66×10^−5^	0.12	0.03	0.01	0.73	4.51×10^−5^	0.06
rs302719	*RERE*	1	8412907	G/T	7.01×10^−5^	0.12	0.03	0.01	0.73	4.84×10^−5^	0.06
rs349171	*PTPRG*	3	62026751	T/C	9.39×10^−5^	−0.16	0.04	0.50	0.08	9.58×10^−5^	0.35
rs8336	*SMARCAD1*	4	95430633	T/C	9.81×10^−5^	−0.13	0.03	1.43×10^−3^	0.18	0.02	0.47

Rows were ordered by *P*_meta_.

### Functional enrichment analysis

Table 2 summarizes the results of pathway enrichment analysis of the 205 module genes by the Ingenuity Pathway Analysis (IPA). Enrichment results of KEGG [15] pathways were shown in Table S1. The enriched pathways included Wnt/β-catenin signaling, CREB signaling in neurons, Calcium signaling, Gα12/13 signaling, and synaptic long term depression. Overall, the results are consistent with the neuropathology and immune/inflammation hypotheses in schizophrenia [24], [25], suggesting that our DMS-based strategy is effective on detecting joint association signals from multiple GWAS datasets.

**Table 2:** Enriched pathways for module genes by Ingenuity Pathway Analysis.

Ingenuity Canonical Pathways	-log(*P*_BH_)	Molecules
Huntington's Disease Signaling	7.17	*GRIN2B, HDAC2, GRB2, CREBBP, HDAC1, GNB2L1, DNM3, ITPR1, POLR2B, SIN3A, EP300, JUN, IGF1, CACNA1B, PRKCE, PIK3CB, PRKCH, EGFR*
Wnt/β-catenin Signaling	6.52	*GJA1, TGFBR3, HDAC1, CREBBP, SOX13, ACVR1B, EP300, MYC, CDH2, CDH1, JUN, CSNK2A1, CTNNB1, ACVR2A, SOX5*
Androgen Signaling	4.97	*JUN, AR, GNA12, GNB2L1, CREBBP, GNAI1, PRKCE, PRKCH, POLR2B, GNA13, EP300*
CREB Signaling in Neurons	4.86	*GRIN2B, GRB2, GNA12, GNB2L1, CREBBP, GNAI1, POLR2B, ITPR1, EP300, PRKCE, PIK3CB, PRKCH, GNA13*
Prolactin Signaling	4.82	*MYC, FYN, JUN, GRB2, CREBBP, PRKCE, PIK3CB, PRKCH, EP300*
TGF-β Signaling	4.40	*JUN, GRB2, HDAC1, CREBBP, SMAD7, SMAD5, ACVR2A, ACVR1B, EP300*
Calcium Signaling	4.31	*GRIN2B, TNNT1, TRPC1, HDAC2, RYR3, RYR2, HDAC1, CREBBP, MYH9, ITPR1, ACTA1, EP300*
Gα12/13 Signaling	4.12	*CDH2, CDH1, JUN, F2R, GNA12, IKBKE, PIK3CB, GNA13, CDH16, CTNNB1*
Synaptic Long Term Depression	3.97	*PRKG1, IGF1, GNA12, RYR3, RYR2, GNAI1, PRKCE, PRKCH, GNA13, ITPR1*
Dopamine-DARPP32 Feedback in cAMP Signaling	3.80	*KCNJ12, PPP1CC, GRIN2B, PRKG1, CREBBP, GNAI1, CACNA1C, PRKCE, DRD5, PRKCH, ITPR1*

*P* values adjusted by Benjamini & Hochberg (BH) method [33].

## Discussion

We proposed a novel strategy to prioritize candidate genes from multiple GWAS datasets in the context of the human interactome and applied it to schizophrenia. Integration of the PPI network and implementation of our dense module search algorithm greatly improved the coverage of gene annotations, introduced gene set flexibility when searching for candidate genes, and allowed for dynamic identification of putative genes. The bidirectional strategy we proposed here made full use of the discovery and evaluation datasets to avoid potentially incomplete discovery using either one of them separately. The final subnetwork and candidate gene list display the combined results of the two processes, namely GAIN (discovery) → ISC (evaluation) and ISC (discovery) → GAIN (evaluation); thus, they are comprehensive and cohesive in revealing the signals from both datasets. At the molecular level, the module genes we identified showed substantial overlap with previous studies. We also identified novel genes that had not been studied in schizophrenia, yet could be promising new candidates.

The procedure we proposed in this study implemented our previously developed dense module search algorithm. One important improvement is that we introduced *P*(*Z_m_*) for module selection, instead of simply relying on the module score, *Z_m_*, although the latter is straightforward and has been proved effective in our previous work [14]. In this study, we adopted the Efron et al. [26] method and computed *P* values based on *Z_m_* scores through the estimation of empirical null distribution. Theoretically, *Z_m_* and the corresponding *P*(*Z_m_*) values are expected to have identical rank, which has also been observed in real data (Spearman correlation coefficient = 1). In contrast to applying a straightforward cutoff value of *Z_m_* to perform module selection, *P*(*Z_m_*) examines the overall distribution of all module scores and has the advantage to provide a statistical evaluation. Thus, we replaced *Z_m_* by *P*(*Z_m_*) for module selection in the current study. Alternatively, using simulated genotyping and phenotype data to estimate the proportions of modules that can capture the most causal variants will help module selection. In such cases, appropriate simulation data for the analyzed disease model is important for both power estimation and module selection, and will be considered in our future work.

One limitation of our method is that the dense module searching process is sensitive to the background network. The algorithm of DMS examines all the neighborhood nodes within the distance of *d* and selects the best node in every step of module expanding. Although this is an advantage to recruit the best node(s) in each step, it also makes the DMS algorithm heavily rely on the searching space defined by the background interactions. Currently, our knowledge of human PPI network is far from complete. To reduce the uncertainty of network data, we required our working network only include interactions with experimental evidence while excluding interactions predicted by computational algorithms. However, because our aim is to search for a subnetwork that is significantly enriched with GWAS signals, the background PPI network can be extended to any network that is built under a rational biological hypothesis, e.g., co-expression network, functional correlated network, or network based on co-occurrence in literature. Using any of these potential datasets, the strategy we proposed here can be easily extended while the aim is always to search for a subnetwork that is significantly enriched with association signals from GWAS data.

We performed meta-analysis using three GWAS datasets, two of which have already been used for module construction. In the latter case, the ISC and GAIN datasets were used at the gene and module levels, while in the meta-analysis, the examination of the three GWAS datasets was conducted at the SNP level, including its mutation direction. The results of meta-analysis were intended to provide a complementary view and further examination of association signals of the module genes at the SNP level rather than in any single GWAS dataset. Of note, an ideal way of replication of the module genes is to test them in other datasets that are completely independent of those having already been used in the module construction step; however, there are only limited number of independent GWAS datasets for schizophrenia at the current stage. To partially accomplish this evaluation goal, we examined the module genes in the nonGAIN dataset, an independent dataset from those (ISC and GAIN) in module selection. The evaluation results of the nonGAIN dataset thus provide some replication evidence of the module genes.

There have been a few previous studies combining network data with GWAS data. A representative method is DAPPLE, which takes the association loci in GWAS datasets as input and tests whether genes located in these loci are significantly connected via PPI. The advantages of DAPPLE include that it does not require the genotyping data of the original GWAS datasets, it provides a comprehensive randomization test to address the high-degree nodes, and it has an online tool for public use. Although DAPPLE and the method we proposed here both use PPI network to analyze GWAS data, they differ substantially in term of the underlying hypothesis. DAPPLE tests whether the associated genes are significantly connected compared to random networks while our method searches for modules that are significantly associated with the disease. Due to this main difference as the starting point, the two methods differ in many aspects in the subsequent analyses, such as the way to build the resultant network and the way to evaluate the results. For example, DAPPLE only takes the associated loci as input, which are typically defined by 5×10^−8^ and all the other loci, including those with weak to moderate association levels, would be discarded. This might be less efficient in searching association modules, especially for diseases or traits that do not have strong association signals from GWAS. For example, for psychiatric diseases such as schizophrenia, association signal of the markers in any single GWAS dataset failed to reach the genome-wide significance level 5×10^−8^. Specifically, if we use DAPPLE to analyze any of the three GWAS datasets used in this study, we would not have any associated loci based on the significance level 5×10^−8^. In contrast, DMS considers all the genes genotyped in the GWAS as input (seeds) in the network, and searches for the final modules in a weight-guided fashion. Here, the weight is from GWAS *P* values. Subsequently, many moderately associated genes (e.g., those with *P* values between 0.05∼5×10^−8^) might have chance to be included in the final modules for an examination of their joint effects. In practice, depending on the purposes of each study and data availability, investigators may choose appropriate methods for their specific testing.

The merged subnetwork (Figure S3) included a number of well-studied candidate genes for schizophrenia, such as *DISC1*, *DLG2*, *DLG3*, *DRD5*, *GNA12*, *GNA13*, and *GNAI1*. Many genes have been studied in previous association studies [2], [3]. Interestingly, *GRB2* was present in the merged network. We identified *GRB2* as a candidate gene for schizophrenia in our previous study through a network-assisted strategy [24] and then validated it in the Irish Case Control Study of Schizophrenia (ICCSS) sample [27]. Here, using an independent strategy and datasets, we again identified this gene, further supporting *GRB2* as a candidate gene for schizophrenia. The canonical pathways enriched in the module genes also confirmed the involvement of neuro-related genes and pathways in schizophrenia.

In summary, we have performed a comprehensive network-based analysis using our DMS-based approach augmented with IPA software to facilitate interpretation. The outcome of this analysis not only supports previously reported associations with schizophrenia, but also implicates functional components such as the Calcium signaling, Gα12/13 signaling, and the synaptic long term depression pathways in schizophrenia risk. Future work to estimate the power of this network-based strategy through simulation and validation in independent samples will enhance the applications of this method in other diseases or traits.

## Materials and Methods

### GWAS datasets

The Genetic Association Information Network (GAIN) dataset for schizophrenia was genotyped using the Affymetrix Genome-Wide Human SNP 6.0 array, and our access to it was approved by the GAIN Data Access Committee (DAC request #4532-2) through the NCBI dbGaP. We used the samples of European ancestry. Quality control (QC) was executed as follows. For individuals, we excluded those with a high missing genotype rate (>5%), extreme heterozygosity rate (±3 s.d. from the mean value of the distribution), or problematic gender assignment. We used PLINK [28] to compute the identify-by-state (IBS) matrix to pinpoint duplicate or cryptic relationships between individuals, and we retained the sample with the highest call rate for each pair of samples with an identity-by-descent (IBD)>0.185. Principle component analysis (PCA) was performed using the smartpca program in EIGENSTRAT [29] to detect population structure and to allow removal of outlier individuals. Eight significant PCs with the Tracy Widom test *P* value<0.05 were then used as covariates for logistic regression (additive model). For genotyped SNPs, we removed those with a missing genotype rate>5%, minor allele frequency (MAF)<0.05, or departing from Hardy-Weinberg equilibrium (*P*<1×10^−6^). The final analytic dataset included 1,158 schizophrenia cases, 1,377 controls, and a total of 654,271 SNPs with a genomic inflation factor λ = 1.04.

The International Schizophrenia Consortium (ISC) samples were collected from eight study sites in Europe and the US [2]. The samples were genotyped using Affymetrix Genome-Wide Human SNP 5.0 and 6.0 arrays, and this data was initially analyzed by ISC [2]. A total of 3,322 patients with schizophrenia, 3,587 normal controls of European ancestry, and 739,995 SNPs were included in our analysis. To account for potential population structure caused by collection sites, we used the Cochran-Mantel-Haenszel test for a single marker association test, following the original report [2].

The Molecular Genetics of Schizophrenia (MGS) - nonGAIN dataset (denoted as “nonGAIN” hereafter) was genotyped in the same laboratory as GAIN, but in different phases. Access to this dataset was approved by dbGaP (DAC request #4533-3). Similar QC and PCA as described for GAIN were performed. This process retained 1,068 cases and 1,268 controls, all of which are of European ancestry, and 623,059 SNPs for subsequent analysis. Fifteen significant PCs with the Tracy-Widom test *P* value<0.05 were used as covariates for logistic regression (additive model) using PLINK, with λ = 1.04.

We mapped SNPs to human protein-coding genes downloaded from NCBI ftp site (Build 36). A SNP was assigned to a gene if it was located within or 20 kb upstream/downstream of the gene [30]. Each gene was assigned a gene-wise *P* value using the *P* value of the gene's most significant SNP. A total of 19,739 genes were successfully mapped in the GAIN dataset and 19,910 in the ISC dataset.

### Human protein-protein interaction (PPI) network

A comprehensive human PPI network was downloaded from the Protein Interaction Network Analysis (PINA) platform [31] (March 4, 2010), which collects and annotates data from six public PPI databases (MINT, IntAct, DIP, BioGRID, HPRD, and MIPS/MPact). To ensure the reliability of the network, we only kept those interactions having experimental evidence and both interactors are human proteins. Our working network included a total of 10,377 nodes (genes) and 50,109 interactions. Only common genes that were represented in both GWAS and PPI datasets were retained for subsequent analysis.

### Dense module search analysis

We applied our recently developed dense module search (DMS) algorithm [14] with substantial improvement to these schizophrenia GWAS datasets. Details of the DMS algorithm are provided in reference [14]. Briefly, DMS works with a node-weighted PPI network and searches for a best module for each node in a score-guided fashion. A quantitative description of the network includes each node weighted by 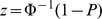, where 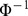 is the inverse normal cumulative density function and *P* is the *P* value representing the association signal in the gene region (which we called the gene-wise *P* value) from the GWAS dataset. Each module is scored by 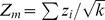, where *k* is the number of nodes (genes) in the module.

Given a single GWAS dataset, we first overlay gene-wise *P* values to the PPI network to generate a GWAS *P* value-weighted working network. We then took each of the nodes in the network as a seed gene, and searched for a best scored module for it. In each case, starting with the seed ‘module’ formed by the seed node, the DMS algorithm searches for the node with the highest score in the neighborhood within a distance *d* (*d* = 2) to the seed module. Then, the module is expanded by adding the highest-scored node if *Z_m+1_*>*Z_m_*×(1+*r*), where *Z_m+1_* is the new module score after adding the node, *Z_m_* is the original module score and *r* is a pre-defined rate. We set *r* to be 0.1 in this study. This module expansion process iterates until none of the neighborhood nodes can satisfy the function *Z_m+1_*>*Z_m_*×(1+*r*). Because this module construction process was conducted taking each node in the network as the seed gene, several thousands of modules are expected corresponding to the thousands of nodes.

### Module assessment

We provided three procedures to assess the significance of the identified modules, each of which aims to build null distributions for different hypotheses.

First, to perform significance test of the identified modules, we calculated *P* values based on module scores (*Z_m_*) for each module by empirically estimating the null distribution [26]. According to Efron et al. (2010), the null distribution is a normal distribution with mean δ and standard deviation σ, both of which can be empirically estimated using the R package *locfdr*. Specifically, module scores were first median-centered by subtracting the median value of *Z_m_* from each of them, followed by estimation of the parameters of δ and σ for the empirical null distribution using *locfdr*. The standardized module scores (*Z_S_*) were then calculated and converted to *P* values, *P*(*Z_m_*) = 1-Φ(*Z_S_*), where Φ is the normal cumulative density function.

Second, to determine whether the module score is higher than expected by chance, a standard way is to randomly select the same number of genes in a module, i.e., resample genes in the network regardless of the interactions, and compare the module score in the random gene set with the score in the real case. Specifically to alleviate the biases in GWAS data (e.g., gene length or SNP density) or the network data (e.g., high-degree nodes), we incorporated weighted resampling which intentionally matches the pattern of biases in each resample to resemble the real case. The gene length bias and the SNP density bias are commonly noticed in GWAS datasets, especially when using the most significant SNP to represent genes [30]. This is because when mapping SNPs to genes, longer genes tend to have more SNPs and in turn have higher chance to be significant. These two types of biases are closely correlated but differ in cases due to different genotyping platforms. For both biases, we first estimate a weight for each gene based on the specific character to be adjusted, and then performed weighted resampling to ensure each of the resample has the similar pattern in term of the adjusted character. This weighted resampling procedure ensures that genes could be selected in a similar pattern of gene length or SNP density as in the real GWAS data. Therefore, the empirical *P* values for each module built on the bias-matched permutation data could be adjusted by gene length (*P*_GL_) or the number of SNPs per gene (*P*_nSNPs_). A detailed description of this function can be found in previous work [23].

Another type of bias was that, in the PPI network, nodes with many interactors (high degree) are more likely to be recruited in module expansion steps. We thus categorized all the nodes in the working PPI network into four categories by their degree values (degree range 0–2^2^, 2^2^–2^4^, 2^4^–2^6^, and >2^6^) (Figure S1). For each module, a topologically matched random module was generated by randomly sampling the same number of nodes in each of the four node bins. An empirical *P* value is computed by 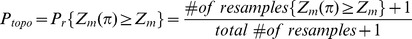, where 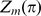 is the score of the random module for the *π* ^th^ resample, and 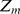 is the observed module score.

Third, to assess the disease association of the modules, we performed permutation test by shuffling case/control labels in the GWAS datasets. We generated 1,000 permutation datasets using the genotyping data, and computed module scores in each permutation dataset in the same way as for the real case. An empirical *P* value for each module was computed according to 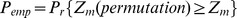, where *Z_m_*(*permutation*) is the module score in the permutation data.

A combinatorial set of criteria was defined to select modules: (1) *P*(*Z_m_*)<0.05; (2) *P*_GL_<0.05, *P*_nSNPs_<0.05, and *P*_topo_<0.05; and (3) *P*_emp_<0.05. This set of combinatorial criteria is applied whenever one GWAS dataset is used to identify, assess and select modules. When there is an additional GWAS dataset available for evaluation, we included two additional criteria: (1) *P*(*Z_m_*_(*eval*)_)<0.05 and/or (2) *P*_emp(eval)_<0.05.

### Meta-analysis

Meta-analysis of module genes was conducted using three major GWAS datasets: ISC, GAIN, and nonGAIN. A quality control step was performed before the meta-analysis to detect whether there is duplication or cryptic relatedness among the samples in the three GWAS datasets. Pairwise IBS was computed using an unrelated list of markers (generated through the option “–indep-pairwise 50 5 0.2” in PLINK [32]). No pair was observed with an IBD>0.185, a cutoff value that is halfway between the expected IBD for third- and second-degree relatives. We performed inverse-variance weighted meta-analysis based on the fixed-effects model using the tool *meta* (http://www.stats.ox.ac.uk/~jsliu/meta.html). This method combines study-specific beta values under the fixed-effects model using the inverse of the corresponding standard errors as weights. Between-study heterogeneity was tested based on I^2^ and Q statistics. SNPs with evidence of heterogeneity were removed.

The three GWAS datasets were genotyped on the same platform; thus, we performed meta-analysis directly on the genotyped SNPs without imputation. Genomic control within each study was conducted in the meta-analysis using the lambda value to adjust the study-specific standard error (SE).

### Functional enrichment tests

We performed pathway enrichment analysis by the IPA system (http://www.ingenuity.com) and also using canonical pathways from the KEGG database [9] by the hypergeometric test. The KEGG pathway annotations were downloaded in March 2011, containing 201 pathways with size ≥10 and ≤250. For each gene set collection, the results by the hypergeometric test were adjusted by the Bonferroni method for multiple testing correction. To further assess the significance of the identified gene sets, we performed empirical assessment of the significance by resampling 1000 times from the network genes, with each resample containing a random set of 205 genes. For a gene set *S*, we recorded the number of resamples in which the gene set was significant and computed an empirical *P* value by 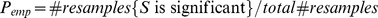.

## Supporting Information

Figure S1 Degree distribution of GAIN GWAS-weighted (top) and ISC GWAS-weighted (bottom) networks. Each node in the network was assigned to a degree bin based on its -log_2_(degree) value.
(PDF)

Figure S2 Module size distribution of GAIN GWAS-weighted (top) and ISC GWAS-weighted (bottom) networks.
(PDF)

Figure S3 Protein-protein interaction network consisting of module genes for schizophrenia.
(PDF)

Table S1 Functional enrichment results using KEGG pathways for module genes.
(DOCX)
