# Supplementary figures and images for: Network-Assisted Investigation of Combined Causal Signals from Genome-Wide Association Studies in Schizophrenia
Source: PLoS Comput Biol. 2012 Jul 5;8(7):e1002587. doi: 10.1371/journal.pcbi.1002587 (PMC3390381; doi:10.1371/journal.pcbi.1002587)

**Degree distribution of GAIN-weighted network**

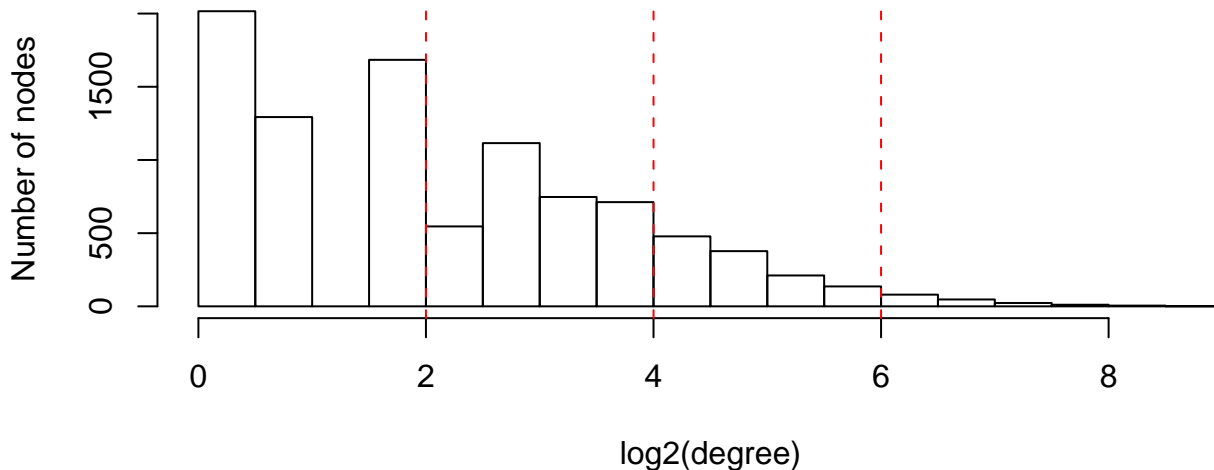

**Degree distribution of ISC-weighted network**

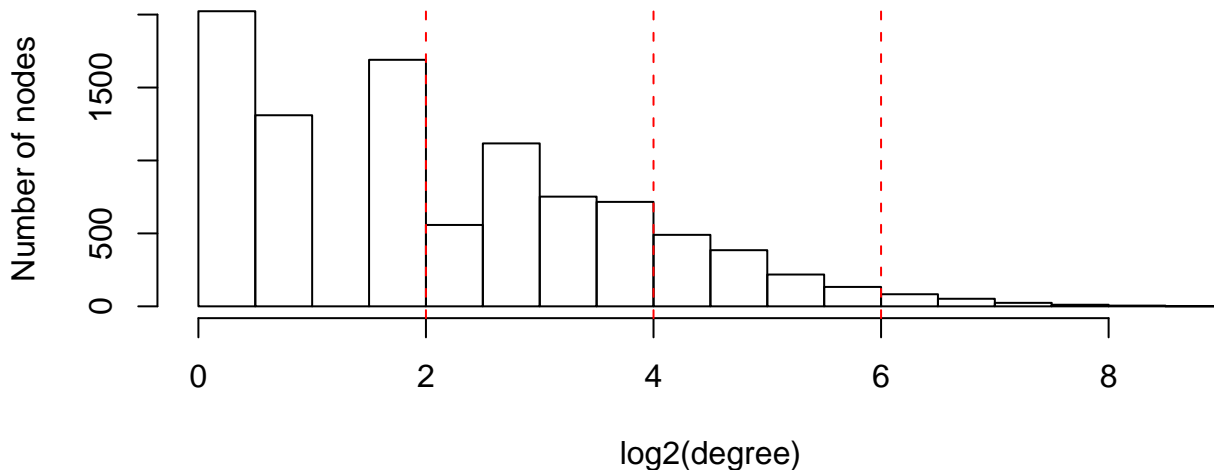

Supplement: Figure S1 — Degree distribution of GAIN GWAS-weighted (top) and ISC GWAS-weighted (bottom) networks. Each node in the network was assigned to a degree bin based on its -log2(degree) value. (PDF) [file pcbi.1002587.s001.pdf]

**GAIN module size**

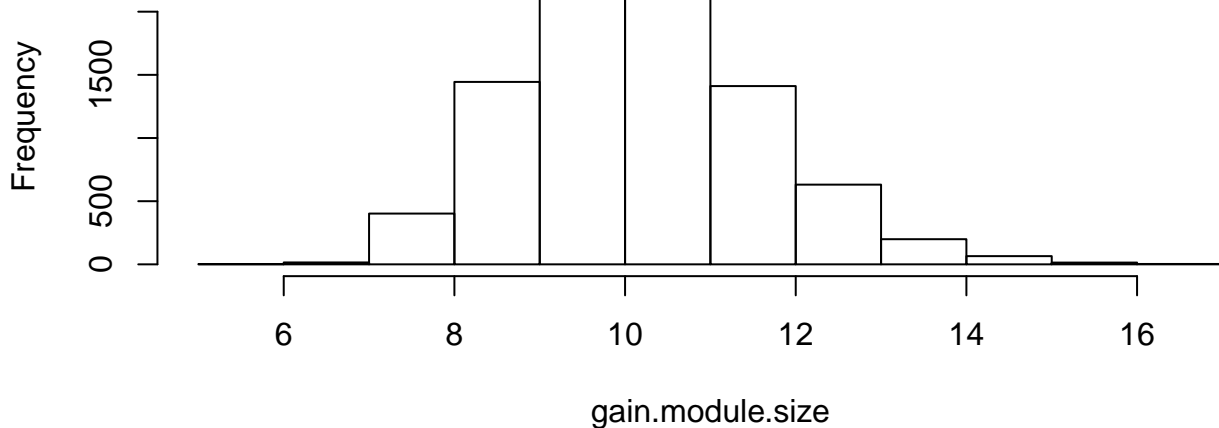

**ISC module size**

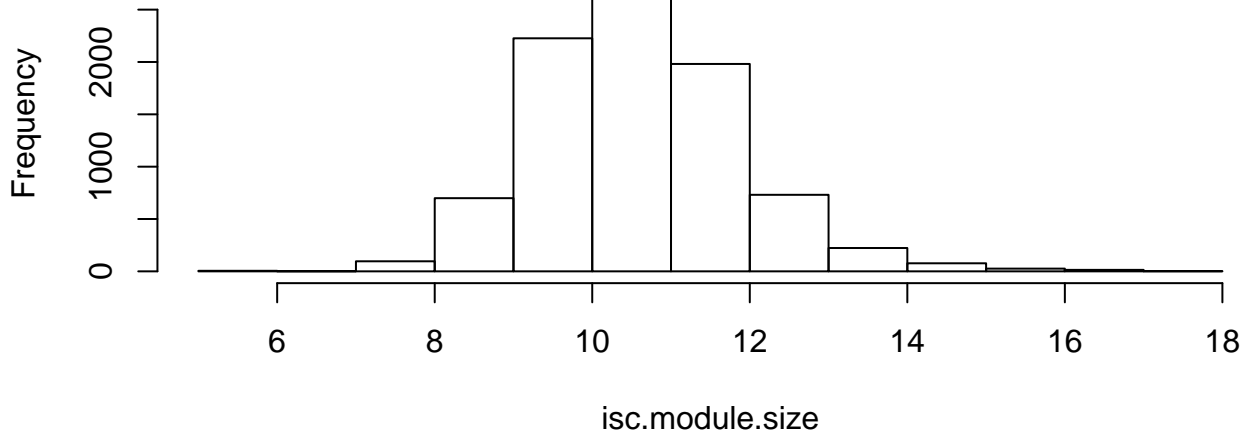

Supplement: Figure S2 — Module size distribution of GAIN GWAS-weighted (top) and ISC GWAS-weighted (bottom) networks. (PDF) [file pcbi.1002587.s002.pdf]

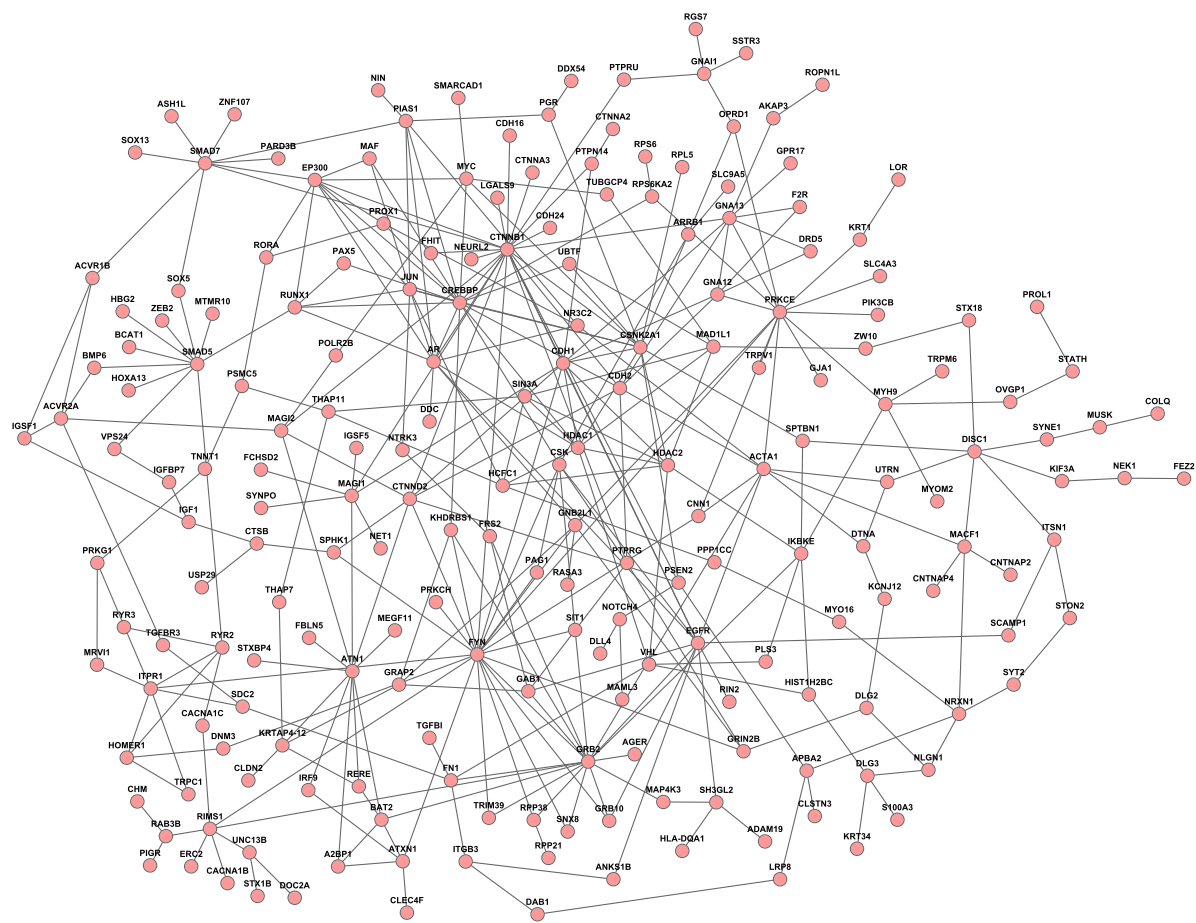

Supplement: Figure S3 — Protein-protein interaction network consisting of module genes for schizophrenia. (PDF) [file pcbi.1002587.s003.pdf]
