# Supplementary material for: Network-Assisted Investigation of Combined Causal Signals from Genome-Wide Association Studies in Schizophrenia
Source: PLoS Comput Biol. 2012 Jul 5;8(7):e1002587. doi: 10.1371/journal.pcbi.1002587 (PMC3390381; doi:10.1371/journal.pcbi.1002587)
Supplement: Table S1 — Functional enrichment results using KEGG pathways for module genes. (DOCX) [file pcbi.1002587.s004.docx]

**Table S1**. Functional Enrichment Results Using KEGG pathways for Module Genes

| **KEGG** | **N^a^** | **O^b^** | ***P*** | ***P*_emp_^c^** | ***P*_BH_^d^** |
| --- | --- | --- | --- | --- | --- |
| hsa04530: Tight junction | 134 | 14 | 7.40×10^-6^ | 0.001 | 8.07×10^-4^ |
| hsa04330: Notch signaling pathway | 47 | 8 | 2.31×10^-5^ | 0.001 | 1.26×10^-3^ |
| hsa05213: Endometrial cancer | 52 | 8 | 4.97×10^-5^ | 0.001 | 1.81×10^-3^ |
| hsa04520: Adherens junction | 75 | 9 | 1.24×10^-4^ | 0.001 | 3.37×10^-3^ |
| hsa05100: Bacterial invasion of epithelial cells | 73 | 8 | 5.64×10^-4^ | 0.003 | 0.012 |
| hsa05412: Arrhythmogenic right ventricular cardiomyopathy (ARVC) | 76 | 8 | 7.41×10^-4^ | 0.001 | 0.013 |
| hsa05215: Prostate cancer | 89 | 8 | 2.10×10^-3^ | 0.002 | 0.026 |
| hsa04720: Long-term potentiation | 70 | 7 | 2.17×10^-3^ | 0.004 | 0.026 |
| hsa05211: Renal cell carcinoma | 70 | 7 | 2.17×10^-3^ | 0.003 | 0.026 |
| hsa04270: Vascular smooth muscle contraction | 116 | 9 | 3.09×10^-6^ | 0.001 | 0.034 |

^a^The total number of genes in the pathway.
^b^The number of module genes observed in the pathway.
^c^The empirical P value of the pathway (see text).
^d^*P* values adjusted by Benjamini & Hochberg (BH) method [1].

Reference

1. Benjamini Y, Hochberg Y (1995) Controlling the false discovery rate: a practical and powerful approach to multiple testing. J Roy Statist Soc Ser B 57: 289-300.
